# Icariin Is A PPARα Activator Inducing Lipid Metabolic Gene Expression in Mice

**DOI:** 10.3390/molecules191118179

**Published:** 2014-11-06

**Authors:** Yuan-Fu Lu, Yun-Yan Xu, Feng Jin, Qin Wu, Jing-Shan Shi, Jie Liu

**Affiliations:** Key Lab for Pharmacology of Ministry of Education, Department of Pharmacology, Zunyi Medical College, Zunyi 563003, China; E-Mails: xuyunyan@126.com (Y.-Y.X.); jinfeng1115@aliyun.com (F.J.); wuqin@zmc.edu.cn (Q.W.); shijs@zmc.edu.cn (J.-S.S.)

**Keywords:** icariin, clofibrate, mouse live, PPARα, Cyp4a14, lipid-lowering effect

## Abstract

Icariin is effective in the treatment of hyperlipidemia. To understand the effect of icariin on lipid metabolism, effects of icariin on PPARα and its target genes were investigated. Mice were treated orally with icariin at doses of 0, 100, 200, and 400 mg/kg, or clofibrate (500 mg/kg) for five days. Liver total RNA was isolated and the expressions of PPARα and lipid metabolism genes were examined. PPARα and its marker genes Cyp4a10 and Cyp4a14 were induced 2-4 fold by icariin, and 4-8 fold by clofibrate. The fatty acid (FA) binding and co-activator proteins Fabp1, Fabp4 and Acsl1 were increased 2-fold. The mRNAs of mitochondrial FA β-oxidation enzymes (Cpt1a, Acat1, Acad1 and Hmgcs2) were increased 2-3 fold. The mRNAs of proximal β-oxidation enzymes (Acox1, Ech1, and Ehhadh) were also increased by icariin and clofibrate. The expression of mRNAs for sterol regulatory element-binding factor-1 (Srebf1) and FA synthetase (Fasn) were unaltered by icariin. The lipid lysis genes Lipe and Pnpla2 were increased by icariin and clofibrate. These results indicate that icariin is a novel PPARα agonist, activates lipid metabolism gene expressions in liver, which could be a basis for its lipid-lowering effects and its beneficial effects against diabetes.

## 1. Introduction

The peroxisome proliferator activated receptor-α (PPAR-α) plays a pivotal role in the regulation of lipid metabolism and fatty acid oxidation, and is beneficial in protecting against metabolic disorders associated with type-II diabetes and obesity [1,2]. PPARα agonists, such as the fibrates, are used to correct dyslipidemia and decrease risks of cardiovascular diseases [2,3]. PPARα-null mice develop obesity and high plasma triglyceride levels [4], and they are more vulnerable to high-fat diet-induced nonalcoholic fatty liver disease (NASH) [5]. PPARα is expressed highly in liver [6] and is a key regulator for lipid metabolism. Pharmacological activation of PPARα has anti-inflammatory activities in liver, adipose, and vascular tissues [7] and PPARα agonists are proposed to be promising candidates for metabolic disorders [2].

Icariin is a flavonol glycoside isolated from Epimedium genus, which has been used in traditional Chinese medicine for thousands of years. The origination and chemical structure of icariin, its antioxidative properties and medicinal applications have been reviewed [8,9]. Our Laboratory has shown that icariin protects against cerebral ischemia/reperfusion injury [10], improves learning and memory deficit in rats induced by aluminum [11], chronic cerebral hypoperfusion [12], and by D-galactose [13]. The beneficial effects of icariin appear to be related to inhibition of beta-amyloid peptide segments [14], and the protection against lipopolysaccharide-induced neuroinflammation [15]. We have recently shown that icariin improves learning and memory functions in APP/PS1 transgenic mice through the stimulation of NO/cGMP signaling [16].

The beneficial effects of icariin on metabolic syndrome are recently recognized [17,18]. In rabbits fed a high-cholesterol diet, icariin reduced the levels of serum total cholesterol and low-density lipoprotein cholesterol [18]. In streptozocin-induced diabetes rats, icariin ameliorates streptozocin-increased activities of lactate dehydrogenase, acid phosphatase, gamma-glutamyltranspeptidase and alpha-glucosidase in the epididymis and reduces serum lipid and fructose levels [17]. Icariin also ameliorates streptozocin-induced rat diabetic retinopathy [19] and nephropathy [20]. More interestingly, icariin has been shown to induce peroxisome proliferator-activated receptor gamma coactivator-1 alpha (PGC-1alpha), peroxisome proliferator-activated receptor alpha (PPARα) during cardiomyocyte differentiation in murine embryonic stem (ES) cells [21]. In our recent studies, we found that icariin is effective in inducing Cyp4a14, a PPARα-target gene in mouse livers [22], which arouses our interest to examine whether icarriin is a PPARα activator. PPARα regulates genes encoding enzymes and transporters of fatty acid oxidation [1,2,3,4,5,6,23]. We further examined the effects of icariin on the mRNAs of lipid metabolic enzyme genes, using PPARα agonist clofibrate as a positive control. The results revealed that icariin is a novel PPARα activator and provides the molecular basis for its beneficial lipid-lowering effects by inducing fatty acid (FA) β-oxidation enzyme genes.

## 2. Results and Discussion

### 2.1. Effect of Icariin on the mRNA of PPARα and Marker Enzymes

**Figure 1:**
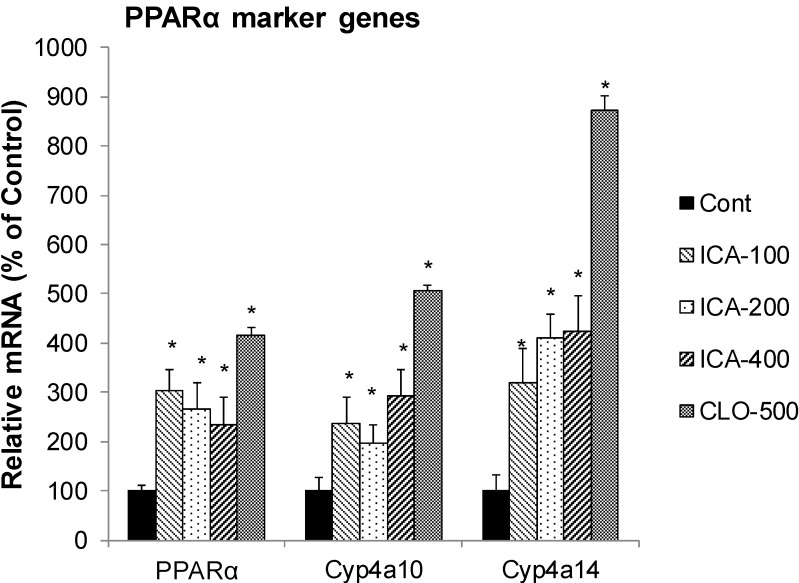
Effects of Icariin (ICA) on expression of PPARα and PPARα marker enzymes. Data are expressed as mean ± SEM (n = 5). * Significantly different from CMC-treated mice (*p* < 0.05).

Figure 1 illustrates the effects of icariin and clofibrate (CLO) on the expression of PPARα and PPARα-target marker genes Cyp4a10 and Cyp4a14. CLO is a well-known PPARα activator, and increased the mRNA of PPARα 4-fold, Cyp4a10 5-fold, and Cyp4a14 8.7-fold. In comparison, icariin also increased PPARα 2.3-fold, Cyp4a10 2.9-fold, and Cyp4a14 4.2-fold. Administration of icarriin and/or CLO did not affect animal body weight, animal general health and did not produce histopathological alterations (Data not shown).

In our preliminary studies, icariin induced Cyp4a14 in Kunming mice [22], and this effect is now further verified in C57BL/6 mice in the present study, with clofibrate as a positive control. The induction of Cyp4a14 suggests that icariin could affect lipid metabolism, as icariin has recently been shown to have lipid-lowering effects in rabbits [18] and in rats [17]. In cultured cells, icariin has been shown to induce PPARα [21]. In the present study, icariin not only induced PPARα, but also PPARα activation marker enzyme gene Cyp4a10 and Cyp4a14. The results indicate that icariin is a novel PPARα activator and provides the molecular basis for its beneficial lipid-lowering effects by inducing FA β-oxidation enzyme genes.

### 2.2. Effect of Icariin on the mRNA of Fatty Acid Binding Protein and Activation

**Figure 2:**
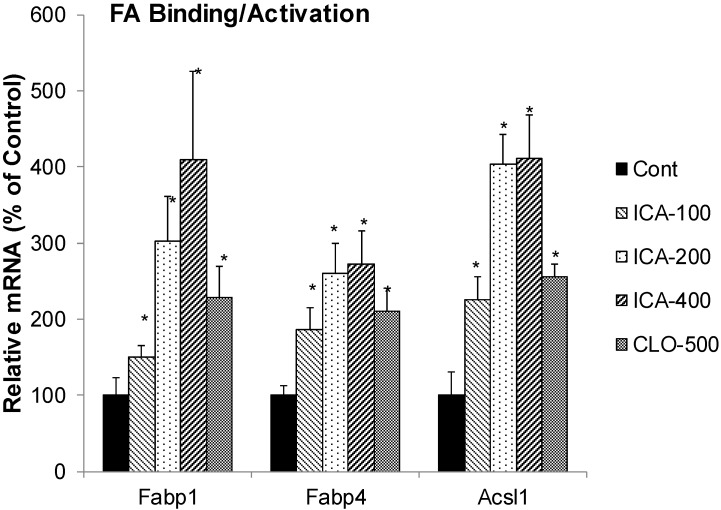
Effects of Icariin (ICA) on expression of fatty acid binding proteins and coactivators. Data are expressed as mean ± SEM (n = 5). * Significantly different from CMC-treated mice (*p* < 0.05).

Figure 2 illustrates the effects of ICA and CLO on the expression of FA binding protein Fabp1, Fabp4 and long chain acyl-CoA synthetase 1 (Acsl1). CLO increased the mRNA of Fabp1 2.2-fold, Fabp4 2.3-fold, and Acsl1 2.6-fold. In comparison, icariin dose-dependently increased Fabp1 4-fold, Fabp4 2.7-fold, and Acsl1 4-fold.

Many drugs could reprogram the liver to adapt various stimuli and maintain homeostasis [24]. For example, PPARα regulates genes encoding enzymes and transporters of fatty acid oxidation [1,2,3,4,5,6,23]. Liver FA binding protein (FABP1) prevents lipotoxicity of free fatty acids and regulates FA trafficking and partition, and is activated by PPARα [25]. FABPs are the major soluble protein that binds very-long-chain n-3 polyunsaturated fatty acids (n-3 PUFAs) in hepatocytes [26]. Mice deficient in liver FA binding protein (L-FABP^-/-^) exacerbated diet-induced weight gain and fat tissue mass gain when fed high-fat diet [27]. Acyl-CoA synthetase is a family of enzymes that catalyze the esterification of FA with CoA. Long-chain acyl CoA synthetase 1 (Acsl1) plays an important role in fatty acid metabolism and triacylglycerol synthesis and increased fatty acid uptake into hepatocytes for esterification [28], and is a PPARα target or co-activator [29]. In the present study the expression of FA binding protein Fabp1 and Fabp4, as well as Acsl1 were increased by icarriin and clofibrate, implying that icarriin activation of PPARα could enhance FA metabolism by increasing FA binding and co-activation.

### 2.3. Effect of Icariin on the mRNA of Mitochondria FA β-Oxidation

**Figure 3:**
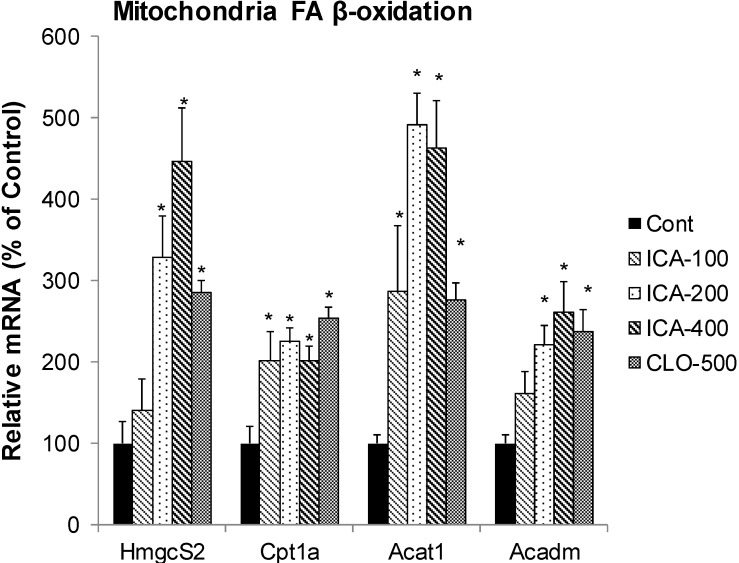
Effects of Icariin (ICA) on expression of mitochondrial fatty acid β-oxidation enzymes. Data are expressed as mean ± SEM (n = 5). * Significantly different from CMC-treated mice (*p* < 0.05).

Figure 3 shows the effects of icariin and CLO on FA mitochondria β-oxidation, including Cpt1a (carnitine palmitoyltransferase 1a, rate-limiting enzyme of mitochondria FA oxidation), Acat1 (acetyl-CoA acetyltransferase 1, an enzyme central to multiple metabolic pathways), Acadm (Acad1) (Acyl-coenzyme A dehydrogenase 1, catalyzes mitochondrial fatty acid beta-oxidation), and Hmgcs2 (Mitochondrial HMG-CoA synthase, the rate-limiting enzyme in ketogenesis). CLO increased the mRNA of Cpt1a 2.8-fold, Acat1 2.5-fold, Acadm 2.4-fold, and Hmgcs2 2.9-fold. In comparison, icariin dose-dependently increased Cpt1a 4.6-fold, Acat1 2.3-fold, Acadm 2.6-fold and Hmgcs2 4.5-fold.

Mitochondria FA β-oxidation and ketone body formation is important in lipid metabolism [30]. Hmgcs2 encodes the key enzyme for ketone-body synthesis, the mitochondrial HMG-CoA synthase [31] and is the rate-limiting enzyme in ketogenesis. Hmgcs2 has been shown to interact with PPARα and acts as a co-activator to up-regulate transcription of genes of lipid metabolism [32]. Cpt1a is a rate-limiting enzyme of mitochondria FA oxidation, which is co-localized with Acsl1 in the liver mitochondrial outer membrane as a part of FA transfer complex [33]. Acat1 is an enzyme central to multiple metabolic pathways [34], and Acadm catalyzes mitochondrial FA β-oxidation [35]. Several compounds such as fenofibrate [35], gemfibrozil analogues [36], inhibitors of FA synthesis [37] and herbal medicine oxymatrine [38], all increase Cpt1a, and/or Acat1, Acadm, Hmgcs2 as a mechanism of their lipid-lowering effects, thus, up-regulation of mitochondrial FA β-oxidation could be a mechanism for lipid lowering effects of icariin.

### 2.4. Effect of Icariin on the mRNA of Peroxisome FA Oxidation

**Figure 4:**
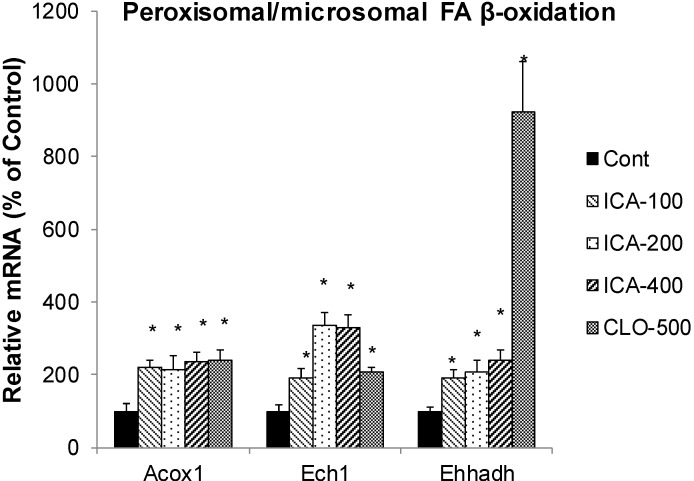
Effects of Icariin (ICA) on expression of peroxisome fatty acid β-oxidation enzymes. Data are expressed as mean ± SEM (n = 5). * Significantly different from CMC-treated mice (*p* < 0.05).

Figure 4 shows the effects of icariin and CLO on peroxisome FA oxidation, including Acox1 (acyl-CoA oxidase 1, a key enzyme involved in peroxisome β-oxidation), Ech1 (enoyl coenzyme A hydratase 1, peroxisome FA oxidation), and Ehhadh (Enoyl-CoA, 3-hydroxyacyl CoA Dehydrogenase, a proximal L-bifunctional protein). CLO increased the mRNA of Acox1 2.4-fold, Ech1 2.1-fold, but markedly increased Ehhadh 11-fold. In comparison, icariin increased Acox1 2.4-fold, Ech1 3.3-fold (in dose-dependent manner), and Ehhadh 2.4-fold.

Peroxisomal FA β-oxidation is another important route of FA metabolism and is regulated by PPARα. Acox1, Ech1, and Ehhadh are key enzymes involved in peroxisomal β-oxidation [39,40,41,42]. Green tea polyphenols [40] and Compound K [42] alleviate obesity by induction of Acox1. Ech1 is an important component of enhanced peroxisomal β-oxidation in C57 mice [39]. Ehhadh is indispensable for the production of medium-chain dicarboxylic acids, providing an explanation for the coordinated induction of mitochondrial and peroxisomal oxidative pathways during fasting [41]. In the present study, the mRNAs of these enzymes were increased by icariin and clofibrate, and marked increase of Ehhadh was seen with clofibrate. The increased peroxisomal FA oxidation together with mitochondrial FA oxidation would enhance FA metabolism and turn over via PPARα activation pathway.

### 2.5. Effect of Icariin on the mRNA of Lipogenesis

**Figure 5:**
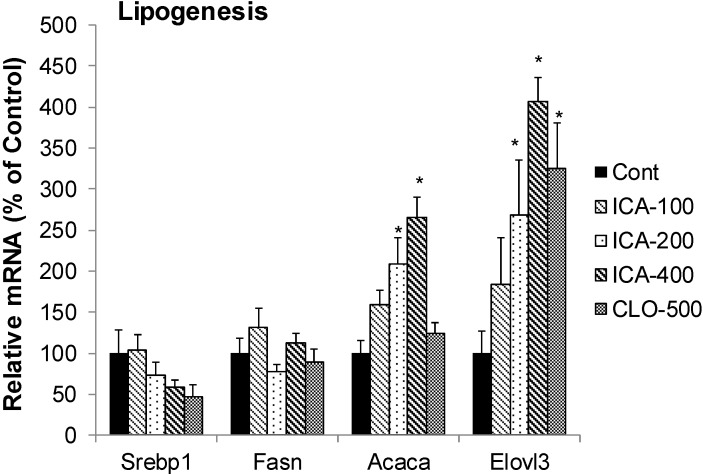
Effects of Icariin (ICA) on Expression of lipogenesis enzymes. Data are expressed as mean ± SEM (n = 5). * Significantly different from CMC-treated mice (*p* < 0.05).

Figure 5 shows the effects of icariin and CLO on mRNAs of lipogenesis enzymes, including Srebp1 (sterol regulatory element-binding protein 1, a transcription factor for lipogenesis), Fasn (fatty acid synthase, a key enzyme in lipogenesis), Acaca (Acetyl-CoA carboxylase 1), a rate-limiting enzyme in FA synthesis, and Elovl3 (elongation of very long chain fatty acids 3, involved in the regulation of the progression of adipogenesis). CLO decreased the mRNA of Srebp1 50%, had no effects on mRNA levels of Fasn and Acaca, but increased Elovl3 3.3-fold. In comparison, icariin also decreased the mRNA of Srebp1 40%, had no effects on mRNA levels of Fasn, but increased the expression of Acaca (2.6-fold) and Elovl3 (4-fold).

Srebp1 is a key transcription factor and Fasn is the key enzyme of lipogenesis [30,43]. The expression of the Elovl3 is under the control of PPARα [44]. Inhibition of Srebp1 and/or Fasn is proposed as a mechanism of lipid-lowering effects of resveratrol [45], betulinic acid [46], and oxymatrine [38]. In the present study, both icariin and clofibrate did not show apparent effects on the mRNA of Srebp1 and Fasn. However, they increased the lipogenesis enzyme Acaca and Elovl3.

### 2.6. Effect of Icariin on the mRNA of Lipolysis

**Figure 6:**
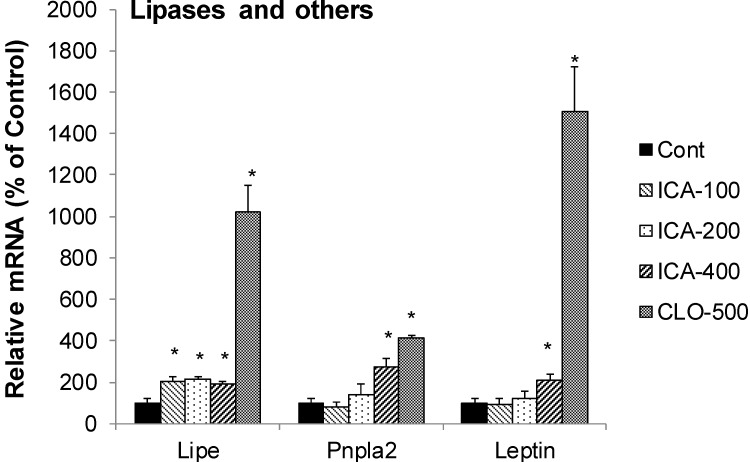
Effects of Icariin (ICA) on expression of lipolytic enzymes. Data are expressed as mean ± SEM (n = 5). * Significantly different from CMC-treated mice (*p* < 0.05).

Figure 6 shows the effects of icariin and CLO on mRNAs of lipolysis enzymes, including Lipe (hormone-sensitive lipase), Pnpla2 (adipose triglyceride lipase, also known as ATGL) and Leptin. CLO increased the mRNA of Lipe 10-fold, Pnpla2 4-fold, but markedly increased the mRNA of leptin (15-fold). In comparison, icariin increased Lipe 2-fold, Pnpla2 (7-fold), and leptin (2-fold).

Another interesting observation in this study is the increased mRNA levels of lipolysis, namely Pnpla2 and Lipe. Lipe is a hormone-sensitive lipase, and Pnpla2 is a members of the Patatin-like Phospholipase Domain containing Protein A (PNPLA) family playing key roles in triglyceride hydrolysis and energy metabolism, particular Pnpla2 (also called ATGL) [47]. Increased expression of Pnpla2 plays a role in protecting steatohepatitis [48]. Thus, the increase of Pnpla2 and Lipe by icariin and clofibrate is related to PPARα activation and would be beneficial and related to their lipid-lowering effects.

## 3. Experimental Section

### 3.1. Chemicals

Icariin was obtained from Zelang Medical Technology CO., LTD (Nanjing, China) with a purity of 98%. Clofibrate was from Sigma (St. Louis, MO, USA) and other chemicals were reagent grade.

### 3.2. Animals and Treatments

Adult 8-week old male C57BL/6 mice were obtained from the Animal Breeding Center (Chongqing, China), and maintained in the SPF-grade animal facilities (Certificate #SYXK2011-004). Mice were acclimated for 1-week in a temperature- and humidity-controlled facility with a standard 12-h light schedule. Mice had free access to SPF-grade rodent chow (Chongqing, China) and purified drinking water. Mice were treated with icariin (100, 200, and 400 mg/kg, dissolved in 2% carboxymethylcellulose sodium (CMC, as a vehicle) for 5 days. Clofibrate (CLO, 500 mg/kg, *po* for 5 days) was used as a positive control, for negative controls, mice were given 2% CMC (10 mL/kg). 24 h after the last dose, livers were collected for analysis. All animal procedures follow the NIH guide of Humane Use and Care Animals, and approved by Institutional Animal Use and Care Committee of Zunyi Medical College.

### 3.3. Animal General Health and Histopathology

Mice were monitored daily, the body weight were recorded. Liver weight was recorded at sacrifice. Liver samples were fixed in 10% formalin prior to routine processing and paraffin embedding. Liver sections (5 µm in thickness) were stained with hematoxylin and eosin and evaluated for hepatocellular necrosis.

### 3.4. RNA Isolation

Total RNA was isolated using RNAiso Plus (TaKaRa Biotechnology, Dalian, China), according to the manufacturer’s protocol. Total RNA was further purified with RNAeasy Mini Kit (Qiagen, Valencia, CA, USA). The concentration of total RNA in each sample was quantified spectrophotometrically at 260 nm. The integrity of each RNA sample was evaluated by formaldehyde-agarose gel electrophoresis and 260/280 nm ratio (>1.8).

### 3.5. Real-Time RT-PCR Analysis

Total RNA in mouse livers was reverse-transcribed into cDNA by High Capacity cDNA Reverse Transcription Kit (Applied Biosystems, Foster City, CA, USA), and the resulting cDNA was used for real-time PCR analysis using Power SYBR^®^ Green PCR Master Mix (Applied Biosystems). Oligonucleotide primers were designed with Primer3 software and listed in Table 1. Relative expression of genes was calculated by the 2-ΔΔCt method and normalizing to the house-keeping gene GAPDH, and the relative transcript levels were calculated as percentage of GAPDH.

### 3.6. Statistical Analysis

Data were analyzed using a one-way ANOVA, followed by Duncan’s multiple range test (*p* ≤ 0.05) utilizing SPSS 13 Software (SAS, Cary, NC, USA). Data are expressed as mean ± SEM.

**Table 1:** Primer sequences for real-time RT-PCR analysis.

Gene	GenBank No.	Forward	Reverse
Acaca	NM_133360	AGAAACCCGAACAGTGGAACT	AGGTAGCCCTTCACGGTTAAA
Acadm	NM_007382	GAGCCCGGATTAGGGTTTAG	TCCCCGCTTTTGTCATATTC
Acat1	NM_009230	TCGATGACTTTGTGACCAACC	TCCACTTCAAACAGCTCGTCT
Acox1	NM_015729	CTTGGATGGTAGTCCGGAGA	TGGCTTCGAGTGAGGAAGTT
Acsl	NM_007981	CAGGCAAAGCATGTCTTCAA	TCCCAGATTTTTGGCTTGTC
Cpt1a	NM_013495	TGATGACGGCTATGGTGTTTC	CAAACAAGGTGATAATGTCCATC
Cyp410	NM_010011	CACACCCTGATCACCAACAG	TCCTTGATGCACATTGTGGT
Cyp4a14	NM_007822	CTGGGTGATGGAACCTCTGT	CATCTGGGAAGGTGACAGGT
Ech1	NM_016772	CGCGATGACAGTTTCCAGTA	TGTGCAGAGGAGCTCAGAGA
Ehhadh	NM_023737	TGGATGTGGATGACATTGCT	GGGGAAGAGTATCGGCTAGG
Elovl3	NM_007703	GAGACCCTGCTTCCCTATCC	CTCCTTCCCTTCATCCTTCC
Fabp1	NM_017399	ATGAACTTCTCCGGCAAGTAC	ACTTTTTCCCCAGTCATGGTC
Fabp4	NM_024406	TCACCTGGAAGACAGCTCCT	AAGCCCACTCCCACTTCTTT
Fasn	NM_007988	GACCTTCATGGACACAATGCT	ATACCACCAGAGACCGTTATG
GAPDH	M32599	AACTTTGGCATTGTGGAAGG	GGATGCAGGGATGATGTTCT
Hmgcs2	NM_008256	CCTCTGTGAATCCTGGGTGT	CTGTGGGGAAAGATCTGCAT
Lipe	NM_010719	ACGCTACACAAAGGCTGCTT	TCGTTGCGTTTGTAGTGCTC
Leptin	NM_008493	CTATGCCACCTTGGTCACCT	ACCAAACCAAGCATTTTTGC
Pnpla2	NM_025802	TCATGTGACACAGCGAGTGA	AAAGAGGAGCCAAGCACAAA
PPARα	NM_011144	GTCCTCAGTGCTTCCAGAGG	GGTCACCTACGAGTGGCATT
Srebp1	AF374266	GTGAGCCTGACAAGCAATCA	GGTGCCTACAGAGCAAGAGG

## 4. Conclusions

The present study clearly demonstrated that icariin is a PPARα activator, induces Cyp4a10 and Cyp4a14, and regulates the mRNA levels of lipid metabolism enzymes and proteins, including fatty acid binding protein, fatty acid oxidation in mitochondria and in peroxisome. Icarriin also had effects on lipolysis and differential effects on lipogenesis, and these effects were similar to well-known PPARα activator clofibrate. These findings are novel in revealing the molecular basis for lipid-lowering effects of icariin in laboratory animals.
